# Improving contraceptive and family planning awareness on a perinatal inpatient unit

**DOI:** 10.1192/j.eurpsy.2021.1468

**Published:** 2021-08-13

**Authors:** G. Xia

**Affiliations:** General Psychiatry, Central and North West London NHS Trust, Wembley, London, United Kingdom

**Keywords:** sexual health, Contraception, Perinatal Psychiatry, Perinatal Mental Health

## Abstract

**Introduction:**

Unplanned pregnancies are a significant risk factor in perinatal mental health. They also have the potential to result in adverse health impacts for mother, baby and children into later in life. Women from disadvantaged backgrounds are less likely to access contraception. Women are more likely to on board health advice during pregnancy and post partum period due to high level of surveillance by health professionals.

**Objectives:**

Our aim was for 90% of patients on Coombe Wood Mother and Baby Unit (MBU) to feel supported to make an informed decision about their contraception by October 2020.

**Methods:**

A questionnaire was completed by fifteen inpatients at the Mother and Baby Unit over a 4 month period (April- August 2020) to assess areas around their pregnancy and contraceptives of choice. Contraceptive training was provided by a Sexual Health Specialist to staff across multiple disciplinaries on Coombe Wood MBU. Sexual Health discussion groups were delivered by doctors to inpatients on a monthly basis. A post-intervention questionnaire was given to patients.

**Results:**

•53% of patients reported unplanned pregnancies. •40% of women felt lacking confidence in choosing the right contraceptive •The most frequent question asked during the sexual health groups was regarding hormonal contraceptives impacting on mental health. •By September 100% of patients felt they were able to make an informed decision about their contraception on discharge.

**Conclusions:**

Facilitating women to make informed decisions regarding their contraception empowers them to gain autonomy, reduces the risks of physical and mental illness, improves the quality of life for mothers and babies.

